# Long-Term Use of Probiotic-Containing Yogurts Is a Safe Way to Prevent *Helicobacter pylori*: Based on a Mongolian Gerbil's Model

**DOI:** 10.1155/2013/594561

**Published:** 2013-11-21

**Authors:** Chao-Hung Kuo, Sophie S. W. Wang, Chien-Yu Lu, Huang-Ming Hu, Fu-Chen Kuo, Bi-Chuang Weng, Chun-Chieh Wu, Chung-Jung Liu, Pei-Yun Tsai, Tsung-Cheng Lee, Li-Wei Chen, Kuang-Hung Cheng, Lin-Li Chang, Deng-Chyang Wu

**Affiliations:** ^1^Division of Gastroenterology, Department of Internal Medicine, Kaohsiung Medical University Hospital, Kaohsiung City 807, Taiwan; ^2^Cancer Center, Kaohsiung Medical University Hospital, Kaohsiung City 807, Taiwan; ^3^Department of Medicine, Faculty of Medicine, College of Medicine, Kaohsiung Medical University, Kaohsiung City 807, Taiwan; ^4^School of Medicine, College of Medicine, I-Shou University, E-Da Hospital, Kaohsiung City 824, Taiwan; ^5^Department of Pathology, Kaohsiung Medical University Hospital, Kaohsiung City 807, Taiwan; ^6^Department of Nursing, Kaohsiung Municipal Hsiao-Kang Hospital, Kaohsiung City 812, Taiwan; ^7^Central Research and Development Institute, Uni-President Enterprises Corporation, Tainan City 710, Taiwan; ^8^Graduate Institute of Biomedical Science, National Sun Yat-Sen University, Kaohsiung City 804, Taiwan; ^9^Department of Microbiology, Kaohsiung Medical University, Kaohsiung City 807, Taiwan; ^10^Department of Internal Medicine, Kaohsiung Municipal Hsiao-Kang Hospital, Kaohsiung City 812, Taiwan

## Abstract

*Background*. The suppression of *Helicobacter pylori* (*H. pylori*) decreases *H. pylori*-related diseases. The probiotics have an inhibitory effect on *H. pylori*. *Aim*. We investigated the effects of long-term use of yogurt on *H. pylori* based on Mongolian gerbils' model. *Materials and Methods*. Yogurt (containing a supplement of *Lactobacillus acidophilus, Bifidobacterium lactis, etc.*) was used. Forty-six gerbils were divided into five groups. All groups were inoculated with *H. pylori* for 5 to 8 weeks. The yogurt was given as follows: Group (Gr.) A: from 1st to 4th week; Gr. B from 5th to 8th week; Gr. C: from 17th week to sacrifice; Gr. D: from 5th week to sacrifice. Gerbils were sacrificed on the 52nd week. Histology was evaluated according to the Sydney system. *Results*. The positive rates of *H. pylori* were 60% (Gr. A), 75% (Gr. B), 67% (Gr. C), 44% (Gr. D), and 100% (Gr. E). Gr. D showed lower inflammatory score. Only Gr. E (60%) had intestinal metaplasia. Gr. D showed higher IL-10 and lower TNF-**α** expression than Gr. E. *Conclusion.* Long-term intake of yogurt could decrease *H. pylori* infection. The long-term use of yogurt would be an alternative strategy to manage *H. pylori* infection.

## 1. Introduction

Currently, *H. pylori *infection is found to correlate with chronic gastritis, peptic ulcer disease, MALT-lymphoma, precancerous changes in the stomach (atrophy and intestinal metaplasia), and stomach cancer. The pathogenic mechanisms leading from chronic active inflammation of the gastric mucosa to the development of ulceration, cancer, and lymphoma remain poorly understood. So it was logical to use animals to survey the possible pathogenesis. Watanabe et al. [1] demonstrated that *H. pylori* infection could induce well-differentiated adenocarcinoma based on a Mongolian gerbil's model. The Mongolian gerbils may represent a useful animal model, thus providing a wonderful opportunity to improve our understanding of the pathogenesis of *H. pylori-*related human gastric disease.

The clinical outcome of *H. pylori* infection is influenced by several factors, including the *H. pylori* strain, the extent of inflammation, and the density of *H. pylori* colonization [2]. Therefore, permanent or long-term suppression of *H. pylori* should decrease the risk of developing *H. pylori*-related diseases [3].

A probiotic is defined as a sufficient number of living microbial species that may have a positive effect to alter the microflora of the host and improve health conditions [4]. Probiotics have been proven to be useful in the treatment of several gastrointestinal diseases such as acute infectious diarrhea [5]. Its effects on *H. pylori-*related gastrointestinal diseases have also raised much interest. Previous studies demonstrated that *Lactobacillus *and* Bifidobacteria* are added to several fermented dairy products and are known to have an inhibitory growth effect on *H. pylori* [6, 7]; however, there is no study surveying the effect of long-term use of these probiotics in an animal model, and whether probiotics ingestion can improve inflammation and atrophy status is debated.

In this study, we investigated the effects of long-term usage of yogurt containing *Lactobacillus acidophilus, Bifidobacterium lactis, Lactobacillus bulgaricus, and Streptococcus thermophiles *on *H. pylori *infection based on a Mongolian gerbil's model.

## 2. Materials and Methods

The experimental design was approved by the Animal Research Committee of Kaohsiung Medical University.

### 2.1. Animals and Housing

8-week-old gerbils with body weight of 30–40 gm were purchased from the Kaohsiung Medical University Experimental Animals Center, Kaohsiung, Taiwan. In usual time, 4 to 5 gerbils per cage were housed and maintained under standard laboratory conditions (room temperature, 23°C~26°C; relative humidity, 55%~65%; 12/12-hour light/dark cycle) with free access to a commercial rodent diet and tap water.

### 2.2. Preparation of the Yogurt

The AB yogurt (President Corp., Tainan, Taiwan), a supplement of *Lactobacillus acidophilus, Bifidobacterium lactis, Lactobacillus bulgaricus, and Streptococcus thermophiles *containing yogurt, was used in this study. The yogurt contains at least 5 × 10^9^ live organisms/200 mL. The yogurt was given mixed with drinking water.

### 2.3. *H. pylori* Inoculation

**Figure 1:**
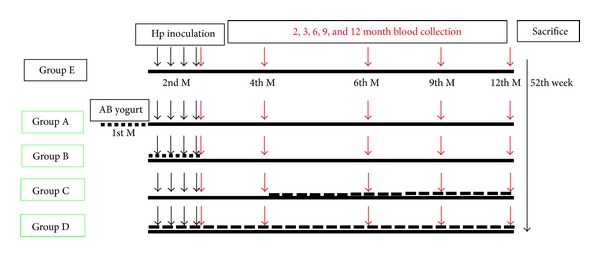
The timing of yogurt given: Gr. A: the yogurt was fed daily in 1st to 4th week. Gr. B: the yogurt was fed from 5th to 8th week. Gr. C: the yogurt was fed since 17th week to the point of sacrifice. Gr. D: the yogurt was fed since 5th week to the point of sacrifice. Gr. E: the yogurt was not given. The animals were sacrificed on the 52th experimental week.

The gerbils were randomly allocated to five groups according to a randomized number. (A–E). All groups were inoculated with *H. pylori *[CagA(+)/VacA(+)] during the 5th to 8th week. The timing of yogurt given was different in Groups A–D. Group A: the yogurt was fed daily in the 1st to 4th week. Group B: the yogurt was fed in the 5th to 8th week. Group C: the yogurt was fed from the 17th week to the point of sacrifice. Group D: the yogurt was fed from the 5th week to the point of sacrifice. Then, all groups were switched to autoclaved distilled water as drinking water. On the 52nd experimental week, the animals were fasted for 24 hours before being sacrificed (Figure 1).

### 2.4. Histological Evaluation of the Gastric Mucosa in *H. pylori*-Infected Gerbils

Samples of the gastric mucosa were excised from each gerbil stomach for the assessment of the presence of *H*. *pylori *and gastric inflammation using Giemsa and hematoxylin-eosin (HE) staining for histological examination, respectively. The samples were fixed in 10% buffered formalin and embedded in paraffin as previous mentioned method [8]. Two experienced pathologists, unaware of the treatment given, performed histological examinations blindly. The specimens were scored according to the updated Sydney system of classification and the grading of gastritis [9]. Histological features of mucosal inflammation and intestinal metaplasia were evaluated for each specimen under a light microscope according to the classification of the Sydney system. The degree of inflammatory cell infiltration and the area of atrophy and intestinal metaplasia were scored as follows: 0, normal; 1, mild; 2, moderate; 3, marked. 

### 2.5. Protein Extraction and Analysis of IL-10 and TNF-*α* Expression in the Gastric Mucosa by Western Blotting

Frozen gastric tissue was homogenized in lysis buffer (100 mmol Tris-HCl, pH 7.4, 15% glycerol, 2 mmol EDTA, 2% SDS, 100 mmol DDT) by the addition of 1 : 20 dilution of aprotinin and 1 : 50 dilution of 100 mmol PMSF. Approximately 100 mg of cellular protein extract was loaded into a well, separated electrophoretically on 13.5% SDS polyacrylamide gel and transferred onto Sequi-Blot TMPVDF membrane (Bio-Rad, Hercules, CA, USA) by electroblotting. Western blotting was performed with polyclonal mouse reactive anti-IL-10 and anti-TNF-*α* were purchased from Santa Cruz Biotechnology, while monoclonal anti-*β*-actin antibody and GAPDH were obtained from Sigma. Visualization of immune complexes was achieved by chemiluminescence using BM Chemiluminescence Blotting Substrate (Boehringer, Mannheim, Germany) and the developed membrane was exposed to an X-ray film (Kodak, Wiesbaden, Germany). Computer-assisted scanning densitometry (Total Lab; Abel) was used to analyze the intensity of the immunoreactive bands.

### 2.6. Stool Collection and Culture for* Bifidobacterium*

We collected approximately 0.2 g fresh stool of gerbils after the yogurt was given for two weeks. The stool samples were stored at 4°C in the refrigerator and transferred for *Bifidobacterium* cultures within 4 hours according to the previous published methods [10]. In brief, a part of wet stool was tested for the percentage of water content in a 65°C vacuum oven. A 3 mL volume of water was added and mixed vigorously with the wet stool by vortex. The tube was then centrifuged at approximately 1000 ×g for 5 minutes. The supernatant was decanted into a clean tube, and the precipitate was washed again. Each supernatant was pooled together. This process was repeated 2–5 times. A serial 10-fold dilution was carried out. For final culture, 0.1 mL of the diluted sample was dispersed on the plates of Center for Disease Control (CDC) agar (Becton Dickinson, Cockeysville, MD, USA) and was incubated anaerobically at 35°C for 48–72 hours. *Bifidobacterium* was identified by colony morphology and Gram stain.

### 2.7. Statistical Analyses

We analyzed the collected data using the statistical software package STATA. Kurskal-Wallis test was used for comparing histological change of mucosa. We also applied the Bonferroni correction for multiple comparisons. An unpaired *t*-test was applied to determine the significance of differences of cytokines expression between the two groups. *P* < 0.05 was considered to be statistically significant.

## 3. Result

There were forty-six gerbils used in this study. The numbers of gerbils in each group were 10 (Group A), 8 (Group B), 9 (Group C), 9 (Group D), and 10 (Group E), respectively. In our study, all gerbils were alive till the end of this experiment; there was no significant difference in the survival rates among the various groups. All gerbils showed gastritis, but there was only one gerbil in Group E with ulcer. We did not find any tumor in all gerbils. All gerbils in Groups A, B, C, and D showed positive results of culture for *Bifidobacterium.*

**Figure 2:**
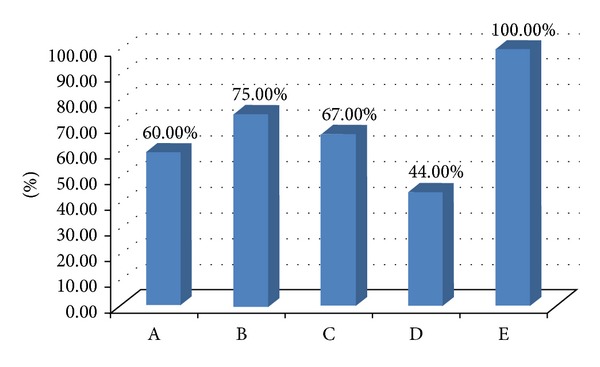
All gerbils in control group (*H. pylori *given only) showed positive result of *H. pylori* test in 52th week. Lower positive rates were noted in those yogurt-fed groups. Group D reveals lowest positive rate. It demonstrated that yogurt can prevent *H. pylori* infection and the effect might be related with the duration of yogurt use.

The success rate of *H. pylori* inoculation was 100% in Group E. On the 52nd week, the positive rates of *H. pylori* were 60% (6/10) (Group A), 75% (6/8) (Group B), 67% (6/9) (Group C), and 44% (4/9) (Group D), respectively (shown in Figure 2). The densities of *H. pylori *were surveyed. It revealed 1.8 ± 0.79 (Gr. A), 2.3 ± 0.70 (Gr. B), 1.6 ± 0.73 (Gr. C), 2.1 ± 0.33 (Gr. D), and 2.8 ± 0.42 (Gr. E). It showed a similar trend as positive rate of *H. pylori* and the lowest density was among Gr. C. It showed that yogurt used in our study could inhibit the growth of *H. pylori *and the effect was significantly obvious in Gr. C (*P* = 0.001, 95% CI: −2.09~−0.40) and Gr. D (*P* = 0.008, 95% CI: −1.82~−0.18).

**Figure 3:**
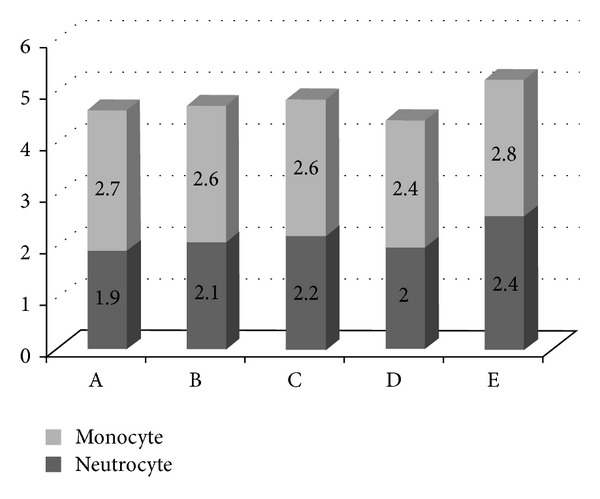
The severities of inflammatory cell infiltration were shown. There was no obvious difference of neutrocyte infiltration among these groups. Similar result was also noted in severities of monocyte infiltration.

We also analyzed the severity of inflammation of gerbil's mucosa according to the Sydney classification. The average severities of neutrocyte infiltration were Gr A: 1.9 ± 0.56, Gr B: 2.1 ± 0.64, Gr C: 2.2 ± 0.44, Gr D: 2.0 ± 0, and Gr E: 2.4 ± 0.70, respectively. The average severities of monocyte infiltration were Gr A: 2.7 ± 0.48, Gr B: 2.6 ± 0.74, Gr C: 2.6 ± 0.53, Gr D: 2.4 ± 0.73, and Gr E: 2.8 ± 0.42, respectively (shown in Figure 3). Gr E showed higher inflammatory score and Gr D showed lower inflammatory score. There was no significant difference between five groups. However, this trend seemed correlated with the positive rates of *H. pylori* in each group.

**Figure 4:**
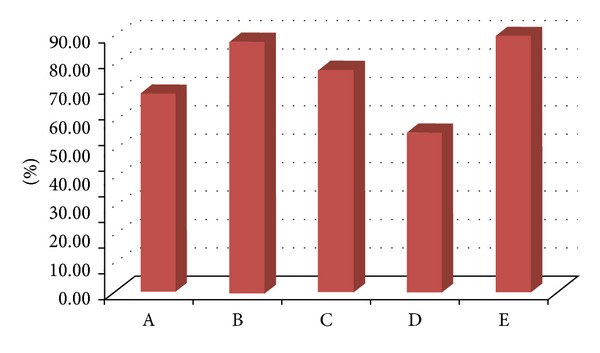
The percentage of obvious atrophy was similar in these groups. However, lower atrophic rate was found in Group D.

We surveyed the severity of atrophy in every group (Figure 4). We regarded the score of atrophy more than 2 as obviously atrophic mucosal change. The percentages of obvious atrophy were Gr A: 70% (7/10), Gr B: 88% (7/8), Gr C: 78% (7/9), Gr D: 56% (5/9), and Gr E 90% (9/10), respectively. The average severities of atrophy were Gr A: 1.9 ± 0.32, Gr B: 1.8 ± 0.35, Gr C: 1.8 ± 0.44, Gr D: 1.56 ± 0.53, and Gr E: 2 ± 0.47, respectively. There was no significant difference found among these groups. Besides this, we did not find intestinal metaplasia in Groups A, B, C, and D. But 60% (6/10) of Gr E had the intestinal metaplasia. It revealed the significant difference (*P* < 0.0001, 95% CI: −1.30~−0.30 ).

**Figure 5:**
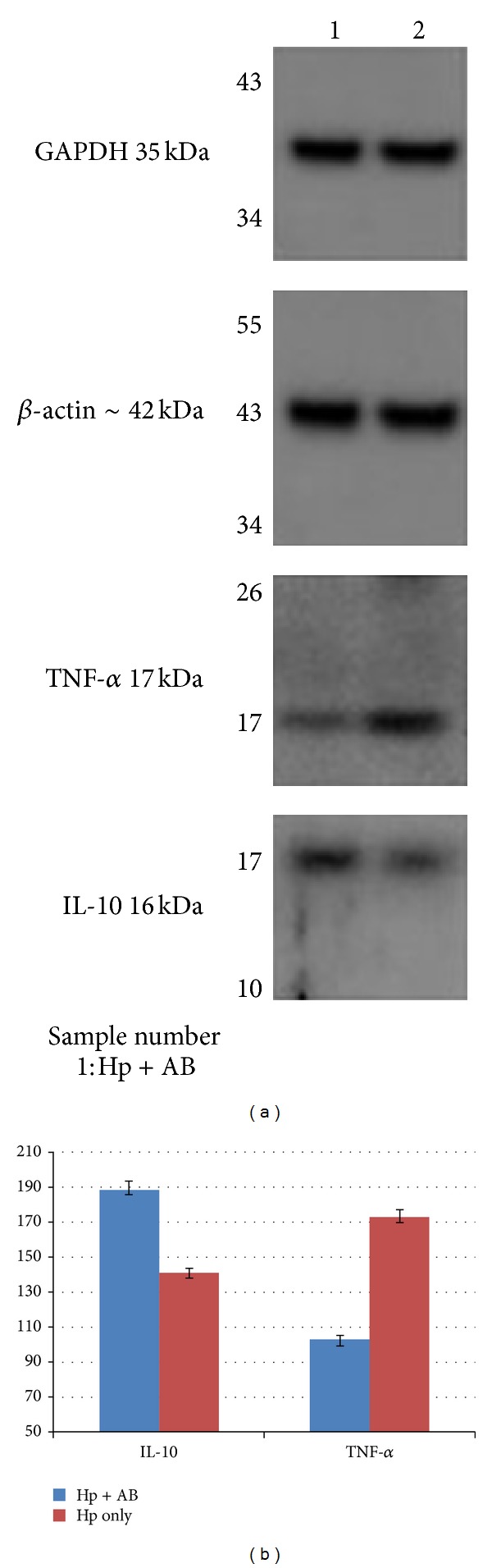
(a) The expression of different proteins in Groups D (AB + Hp) and E (Hp only) by Western blotting. (b) The different protein levels in yogurt-fed (Group D, Hp + AB) and control (Group E, Hp only) groups were shown. Data presented with mean ± SE. Two groups showed similar expression of GAPDH and *β*-actin. We found that Group D had obvious higher IL-10 level (*P* = 0.0002) but lower TNF-*α* level (*P* = 0.0002). Hp: *Helicobacter pylori*, AB: AB yogurt, IL-10: interleukin-10, TNF-*α*: tumor necrosis factor-alpha.

In order to survey the possible mechanism of yogurt's effect, we perform Western blotting for two groups (Gr. D: the longest use of yogurt and Gr. E: no use of yogurt) (Figures 5(a) and 5(b)). Gr. D showed a significant higher level of IL-10 than Gr. E (188.6 ± 4.62 versus 141.0 ± 2.75, *P* = 0.0002). But Gr. D revealed an obvious lower expression of TNF-*α* than Gr. E (102.8 ± 2.57 versus 173.2 ± 3.70, *P* = 0.0002).

## 4. Discussion

Many studies have shown the effects of probiotics resulting in decreased inflammation [11, 12]. However, these previous studies did not survey the long-term effect of probiotics on *H. pylori.* In our data, we firstly showed the beneficial effects of long-term usage of probiotics containing yogurt on decreasing severity of chronic gastritis related with *H. pylori* infection.

The risk of developing *H. pylori*-associated diseases may increase with an increasing level of *H. pylori* density [13, 14]. We also found that the yogurt can led to a decrease in the *H. pylori* load and it was similar to previous studies [15–18]. So it might be reasonable to suppose that yogurt could decrease the incidence of *H. pylori*-related gastrointestinal disease. In our study, we found that longer duration of yogurt use related to more obvious effect of decreasing density of *H. pylori*.

In previous studies, the administration of probiotics alone does not lead to the eradication of *H. pylori*. However, our data showed that groups treated with yogurt showed lower positive rates of *H. pylori* than did the control group (Gr E). In our study, we found that longer duration of yogurt use related to lower positive rates of *H. pylori*. Besides this, it also revealed a trend that earlier consumption of yogurt might have some role in inhibition or eradication of *H. pylori*.

In general, giving probiotics to kids is not harmful, but there is not a lot of proof that it does much good either. So we designed Groups A, B, and D which were the model mimic probiotics used in little children. Groups A and B in our study mimicked the model set by Sakamoto et al. [19]. They disclosed that probiotic-containing yogurt can offer benefits to restore *Bifidobacterium* spp./*E. coli* ratio in children and suppress the *H. pylori* load in *H. pylori*-infected children. Our study also demonstrated a similar finding that the effect of inhibiting *H. pylori* was more obvious in Gr A than in Gr B. This evidence showed a trend that the earlier yogurt is used, the more obvious benefit on *H. pylori* suppression is found. Furthermore, Group D represented probiotics long-term used on little children, so it is worthwhile encouraging parents to give yogurt to their children.

The gastric mucosal barrier is the first line of defense against pathogenic bacteria. Our study revealed that Groups A, B, C, and D showed the obvious effect on decreasing the severity of inflammation. The mechanism of probiotics decreasing the severity of gastric mucosal inflammation is still unclear. It has been suggested that the intake of probiotics strengthens this barrier by producing antimicrobial substances, competing with* H. pylori* for adhesion receptors, stimulating mucin production, and stabilizing the gut mucosal barrier. Certain lactobacilli are resistant to the low pH of the stomach and may adhere to and transiently reside in the human stomach [10, 20]. However, we did not find the residual bacteria in the gerbils' stomach.

Several studies using murine models have shown that treatment with different Lactobacillus strains reduced *H. pylori* or H. felis colonization and decreased Helicobacter-induced gastric inflammation [21–26]. Reduction of *H. pylori* density and gastric inflammation was also observed in specific germ-free mice treated with *L. casei* strain Shirota [23]. However, these models had some limitations. One is that the treating period was not long-term, so they could not confirm the safety and effectiveness of long-term use. Cases using probiotics with serious infection have occurred in those who are immune compromised and have been reported in the literature [27–35], so we also monitored this possible side effect. Fortunately, there was no evidence of severe side effects noted during this study. Another is that the Mongolian gerbil's model is a more suitable model for surveying *H. pylori*-related clinical outcome even carcinogenesis, as other animal models did not disclose similar results. In our study, our gerbil's model demonstrated that long-term use of yogurt was safe and effective in decreasing inflammation by inhibiting the growth of *H. pylori*. So we suggest that the Mongolian gerbil is a reliable model for surveying long-term effect of probiotics on *H. pylori*-related gastrointestinal diseases. In further study, the carcinogen might be added in these models in order to survey the protective effect of probiotics on carcinogenesis of gastric cancer.

In most studies, the effect of probiotics treatment on the level of *H. pylori* infection was estimated indirectly by the urea breath test (UBT) [15–17, 36, 37]. In our study, we used histological findings as the standard of evaluation. We thought that this method could reflect the true effect of yogurt.

Previous animal studies showed that the probiotics effects of lactic acid bacteria may be mediated through immune regulation, particularly through controlling the balance of proinflammatory and antiinflammatory cytokines, which would then result in a reduction of gastric activity and inflammation [38, 39]. However, these previous studies were all short-term models. Our study revealed important information about the impact of long-term probiotics use on changes of cytokines. In our study, the long-term use of probiotics would result in increase expression of IL-10 and decrease of TNF-*α*. These changes would decrease the inflammation of gastric mucosa. It might be the cause of less severe inflammation of gerbil's gastric mucosa in yogurt-fed groups.

The limitation is that we had no reference about whether the amount of probiotics used for gerbils in our study was optimal or not. Besides this, the effect of probiotics on the immune response is difficult to generalize. Distinct probiotics strains may generate divergent immune responses, which, in turn, depend on the host's immune status [40]. The gut microbiota and immunity development in children are different from those in adults. This might be the reason that Groups A and B had the obvious benefit.

In summary, our study supports the effect that long-term intake of products containing probiotics strains, namely, lactobacilli species, can prevent *H. pylori* infection in Mongolian gerbils. It provided the important suggestion that long-term use of yogurt would be a safe and effective strategy for human to prevent* H. pylori* infection and yogurt should be used as early as possible.

## References

[1] Watanabe T, Tada M, Nagi H, Sasaki S, Nakao M (1998). *Helicobacter pylori* infection induces gastric cancer in Mongolian gerbils. *Gastroenterology*.

[2] Ernst PB, Gold BD (2000). The disease spectrum of *Helicobacter pylori*: the immunopathogenesis of gastroduodenal ulcer and gastric cancer. *Annual Review of Microbiology*.

[3] Blaser MJ (1999). Hypothesis: the changing relationships of *Helicobacter pylori* and humans: implications for health and disease. *Journal of Infectious Diseases*.

[4] Fuller R (1991). Probiotics in human medicine. *Gut*.

[5] Gill HS, Guarner F (2004). Probiotics and human health: a clinical perspective. *Postgraduate Medical Journal*.

[6] Wang K-Y, Li S-N, Liu C-S (2004). Effects of ingesting *Lactobacillus*- and *Bifidobacterium*-containing yogurt in subjects with colonized *Helicobacter pylori*. *American Journal of Clinical Nutrition*.

[7] Sheu B-S, Wu J-J, Lo C-Y (2002). Impact of supplement with * Lactobacillus*- and *Bifidobacterium*-containing yogurt on triple therapy for *Helicobacter pylori* eradication. *Alimentary Pharmacology and Therapeutics*.

[8] Kuo C-H, Hu A-M, Tsai P-Y (2009). Short-term Celecoxib intervention is a safe and effective chemopreventive for gastric carcinogenesis based on a Mongolian gerbil model. *World Journal of Gastroenterology*.

[9] Dixon MF, Genta RM, Yardley JH (1996). Classification and grading of Gastritis: the updated Sydney system. *American Journal of Surgical Pathology*.

[10] Yang YJ, Sheu BS (2012). Probiotics-containing yogurts suppress *Helicobacter pylori* load and modify immune response and intestinal microbiota in the *Helicobacter pylori*-infected children. *Helicobacter*.

[11] Sheu B-S, Cheng H-C, Kao A-W (2006). Pretreatment with *Lactobacillus*- and *Bifidobacterium*-containing yogurt can improve the efficacy of quadruple therapy in eradicating residual *Helicobacter pylori* infection after failed triple therapy. *American Journal of Clinical Nutrition*.

[12] Felley CP, Corthésy-Theulaz I, Blanco Rivero J-L (2001). Favourable effect of an acidified milk (LC-1) on *Helicobacter pylori* gastritis in man. *European Journal of Gastroenterology and Hepatology*.

[13] Pantoflickova D, Corthésy-Theulaz I, Dorta G (2003). Favourable effect of regular intake of fermented milk containing *Lactobacillus johnsonii* on *Helicobacter pylori* associated gastritis. *Alimentary Pharmacology and Therapeutics*.

[14] Yamaoka Y, Kodama T, Kita M, Imanishi J, Kashima K, Graham DY (1999). Relation between clinical presentation, *Helicobacter pylori* density, interleukin 1*β* and 8 production, and cagA status. *Gut*.

[15] Tokunaga Y, Shirahase H, Hoppou T, Kitaoka A, Tokuka A, Ohsumi K (2000). Density of *Helicobacter pylori* infection evaluated semiquantitatively in gastric cancer. *Journal of Clinical Gastroenterology*.

[16] Michetti P, Dorta G, Wiesel PH (1999). Effect of whey-based culture supernatant of Lactobacillus acidophilus (johnsonii) La1 on *Helicobacter pylori* infection in humans. *Digestion*.

[17] Gotteland M, Cruchet S (2003). Suppressive effect of frequent ingestion of *Lactobacillus johnsonii* La1 on *Helicobacter pylori* colonization in asymptomatic volunteers. *Journal of Antimicrobial Chemotherapy*.

[18] Linsalata M, Russo F, Berloco P (2004). The influence of Lactobacillus brevis on ornithine decarboxylase activity and polyamine profiles in *Helicobacter pylori*-infected gastric mucosa. *Helicobacter*.

[19] Sakamoto I, Igarashi M, Kimura K, Takagi A, Miwa T, Koga Y (2001). Suppressive effect of *Lactobacillus gasseri* OLL 2716 (LG21) on *Helicobacter pylori* infection in humans. *Journal of Antimicrobial Chemotherapy*.

[20] Conway PL, Kjelleberg S (1989). Protein-mediated adhesion of *Lactobacillus fermentum* strain 737 to mouse stomach squamous epithelium. *Journal of General Microbiology*.

[21] Marteau P, Minekus M, Havenaar R, Huis In’t Veld JHJ (1997). Survival of lactic acid bacteria in a dynamic model of the stomach and small intestine: validation and the effects of bile. *Journal of Dairy Science*.

[22] Aiba Y, Suzuki N, Kabir AMA, Takagi A, Koga Y (1998). Lactic acid-mediated suppression of *Helicobacter pylori* by the oral administration of *Lactobacillus salivarius* as a probiotic in a gnotobiotic murine model. *American Journal of Gastroenterology*.

[23] Sgouras D, Maragkoudakis P, Petraki K (2004). In vitro and in vivo inhibition of *Helicobacter pylori* by *Lactobacillus casei* strain Shirota. *Applied and Environmental Microbiology*.

[24] Coconnier M-H, Lievin V, Hemery E, Servin AL (1998). Antagonistic activity against Helicobacter infection in vitro and in vivo by the human *Lactobacillus acidophilus* strain LB. *Applied and Environmental Microbiology*.

[25] Kabir AMA, Aiba Y, Takagi A, Kamiya S, Miwa T, Koga Y (1997). Prevention of *Helicobacter pylori* infection by lactobacilli in a gnotobiotic murine model. *Gut*.

[26] Johnson-Henry KC, Mitchell DJ, Avitzur Y, Galindo-Mata E, Jones NL, Sherman PM (2004). Probiotics reduce bacterial colonization and gastric inflammation in *H. pylori*-infected mice. *Digestive Diseases and Sciences*.

[27] Sgouras DN, Panayotopoulou EG, Martinez-Gonzalez B, Petraki K, Michopoulos S, Mentis A (2005). *Lactobacillus johnsonii* La1 attenuates *Helicobacter pylori*-associated gastritis and reduces levels of proinflammatory chemokines in C57BL/6 mice. *Clinical and Diagnostic Laboratory Immunology*.

[28] Boyle RJ, Robins-Browne RM, Tang MLK (2006). Probiotic use in clinical practice: what are the risks?. *American Journal of Clinical Nutrition*.

[29] Kunz AN, Noel JM, Fairchok MP (2004). Two cases of Lactobacillus bacteremia during probiotic treatment of short gut syndrome. *Journal of pediatric gastroenterology and nutrition*.

[30] Thompson C, Mccarter YS, Krause PJ, Herson VC (2001). *Lactobacillus acidophilus* sepsis in a neonate. *Journal of Perinatology*.

[31] Broughton RA, Gruber WC, Haffar AAM, Baker CJ (1983). Neonatal meningitis due to *Lactobacillus*. *Pediatric Infectious Disease*.

[32] Perapoch J, Planes AM, Querol A (2000). Fungemia with *Saccharomyces cerevisiae* in two newborns, only one of whom had been treated with ultra-levura. *European Journal of Clinical Microbiology and Infectious Diseases*.

[33] Salminen MK, Rautelin H, Tynkkynen S (2004). *Lactobacillus bacteremia*, clinical significance, and patient outcome, with special focus on probiotic *L. rhamnosus* GG. *Clinical Infectious Diseases*.

[34] Kalima P, Masterton RG, Roddie PH, Thomas AE (1996). *Lactobacillus rhamnosus* infection in a child following bone marrow transplant. *Journal of Infection*.

[35] Soleman N, Laferl H, Kneifel W (2003). How safe is safe? A case of *Lactobacillus paracasei* ssp. paracasei endocarditis and discussion of the safety of lactic acid bacteria. *Scandinavian Journal of Infectious Diseases*.

[36] Salminen MK, Tynkkynen S, Rautelin H (2002). *Lactobacillus bacteremia* during a rapid increase in probiotic use of *Lactobacillus rhamnosus* GG in Finland. *Clinical Infectious Diseases*.

[37] Cats A, Kuipers EJ, Bosschaert MAR, Pot RGJ, Vandenbroucke-Grauls CMJE, Kusters JG (2003). Effect of frequent consumption of a *Lactobacillus casei*-containing milk drink in *Helicobacter pylori*-colonized subjects. *Alimentary Pharmacology and Therapeutics*.

[38] Gill HS (2003). Probiotics to enhance anti-infective defences in the gastrointestinal tract. *Bailliere’s Best Practice and Research in Clinical Gastroenterology*.

[39] Wendakoon CN, Thomson ABR, Ozimek L (2002). Lack of therapeutic effect of a specially designed yogurt for the eradication of *Helicobacter pylori* infection. *Digestion*.

[40] Murosaki S, Muroyama K, Yamamoto Y, Yoshikai Y (2000). Antitumor effect of heat-killed *Lactobacillus plantarum* L-137 through restoration of impaired interleukin-12 production in tumor-bearing mice. *Cancer Immunology Immunotherapy*.

